# Dynamic profiling of bioactive compounds, flavor metabolites, and quality-related microorganisms during the freshening-drying-aging process of citri reticulatae pericarpium: Implications for quality formation mechanisms

**DOI:** 10.1016/j.fochx.2025.102906

**Published:** 2025-08-08

**Authors:** Yuan Hu, Dailing Hu, Lei Yin, Zhi Deng, Yingying Cheng, Hanxi Li, Fu Wang, Youping Liu

**Affiliations:** a State Key Laboratory of Southwestern Chinese Medicine Resources, School of Pharmacy, Chengdu University of Traditional Chinese Medicine, Chengdu 611137, Sichuan, China; b Department of Pharmacy, Shizuishan Maternal and Child Health Hospital, Shizuishan 75300, Ningxia, China

**Keywords:** Citri reticulatae pericarpium, Freshening-drying-aging, Active ingredients, Flavor substances, Quality-related microorganisms

## Abstract

This study systematically explored the dynamic evolution of bioactive components, flavor metabolites, and microbial communities in citri reticulatae pericarpium (CRP) throughout its processing from fresh materials through drying to controlled aging. Using UPLC, GC–MS, and high-throughput sequencing, we found that during the fresh-to-dry phase (0–7 days), water activity decreased significantly (*p* < 0.05), while total flavonoids, hesperidin, and volatile oil yield increased by 3.3 %–10.8 %. Drying at 30 °C enhanced microbial diversity (*p* < 0.05). In the dry-to-aged phase (7–360 days), total flavonoids accumulated, while volatile oil decreased by 50.7 %. *Aspergillus* emerged as a dominant species from 270 to 360 days. Correlation analysis showed positive links between *Aspergillus* and flavonoid levels (*p* < 0.05). This research provides a framework for monitoring CRP processing and highlights the impact of drying methods on quality through microbial diversity.

## Highlights

• The quality of CRP is formed through freshening-drying-aging process.
• Compared the effects of 5 drying methods on the fungal diversity of CRP.
• CRP significantly enhanced fungal diversity under drying conditions at 30 °C.
• *Wallemia* and *Aspergillus* are quality-related microorganisms for CRP.
• The 270 to 360-day aging period is crucial for CRP quality.

## Graphical abstract

**Figure:**
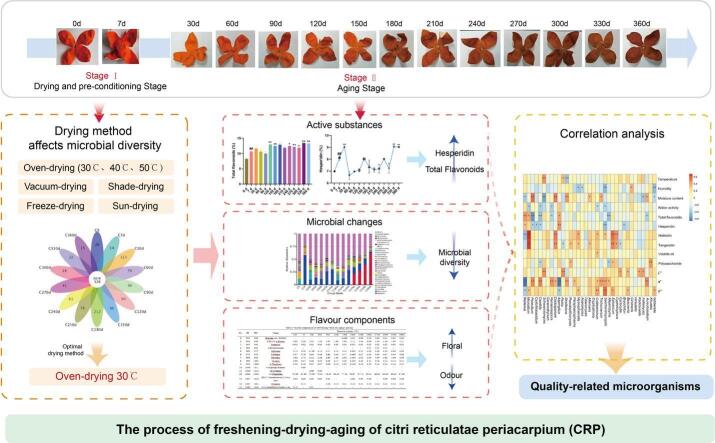

## 1 Introduction

Citri reticulatae pericarpium (CRP), as a typical representative of traditional Chinese resources that are both edible and medicinal, is the mature dried peel of the *Rutaceae* plant *Citrus reticulata* Blanco and its cultivated varieties (Dai et al., 2023). It has a long history of medicinal and edible use. Characterized by its complex chemical composition including flavonoids, volatile oils, alkaloids, and polysaccharides (Yi et al., 2007; X. Yu et al., 2018). CRP is widely utilized for treating digestive and respiratory disorders. Modern research has further revealed its antioxidant, anti-inflammatory, antitumor, and gut microbiota-modulating properties, positioning it as a promising candidate for functional food development and disease prevention (Song et al., 2022; Y. Wang et al., 2022; Zou et al., 2022). Distinct from other herbs or food products, CRP's unique quality formation relies on a dynamic three-phase process: fresh pericarp, initial drying, and prolonged aging (Bian et al., 2022; Huang et al., 2024). Thus, systematically elucidating the transformation patterns of bioactive and flavor components across the freshening-drying-aging continuum, alongside identifying quality-associated microorganisms, is critical for unraveling CRP's quality formation mechanisms and optimizing its processing protocols.

The evolution of food quality is intrinsically linked to processing stages, where transitions from fresh materials to dried intermediates and aged products are accompanied by significant physicochemical and microbial changes (Hanley-Cook et al., 2023). For instance, dehydration concentrates phenolic compounds in raisins but accelerates browning during storage via Maillard reactions (Chen et al., 2022). Pu-erh tea undergoes microbial-driven degradation of polyphenols and synthesis of theabrownins through sun-drying, pile-fermentation, and aging, yielding its characteristic aroma (Li, Zhang, et al., 2022). Similarly, Italy's Parma ham develops rich flavor peptides and volatile aldehydes/ketones through years of enzymatic and microbial synergy during air-drying (Qin, Li, et al., 2024; Qin, Zhao, et al., 2024). These examples underscore the decisive role of processing stages in shaping final product quality. As a representative freshening-drying-aging product, CRP follows analogous mechanisms: fresh pericarp is sun- or oven-dried to reduce moisture, followed by natural aging. The freshening-drying phase involves appropriate temperature drying to eliminate excess moisture, inhibit microbial growth, and stabilize the condition of the peel. This is succeeded by the drying-aging phase, during which the dried peel is stored for an extended period (years or more) in cool, dry, and dark conditions. This prolonged storage allows for continuous and gradual oxidative transformations and concentration of compounds, ultimately resulting in the development of the characteristic complex aroma, rich color, and enhanced medicinal properties that are essential for CRP. During the freshening-drying-aging process, flavonoids gradually transform into more bioavailable aglycones (e.g., hesperetin), while volatile monoterpenes oxidize or cyclize into complex aroma compounds (e.g., guaiazulene, citronellol), ultimately forming CRP's hallmark “brownish color, mellow aroma, and bitter-sweet taste” (Chen, Chen, et al., 2024; F. Yang et al., 2022; P. Yu et al., 2024). Although prior metabolomic studies have mapped compositional shifts during CRP aging and hinted at microbial contributions to flavonoid transformation (Q. Wang et al., 2024), existing research predominantly focuses on isolated timepoints in the aging phase, neglecting dynamic chemical changes and microbial colonization patterns during the critical freshening-drying phase. This fragmented perspective impedes a holistic understanding of the continuity and stage-specific regulation underlying CRP's quality formation, particularly the unexplored “pre-regulatory” effects of drying methods on microbial communities and their cascading impacts on aging.

This study addresses these gaps by integrating multi-omics analyses to systematically investigate the dynamic evolution of bioactive components, flavor metabolites, and fungal communities across CRP's entire freshening-drying-aging processing chain. Using UPLC and GC–MS, we quantified temporal changes in flavonoids and volatile oils, identifying key chemical markers. High-throughput sequencing (Illumina MiSeq) elucidated fungal succession patterns and screened functional microbes correlated with hesperetin accumulation and aroma formation. Spearman correlation analysis further deciphered intrinsic linkages between chemical constituents and microbial communities. By establishing a comprehensive framework spanning CRP's entire processing continuum, this work fills critical knowledge gaps in the understudied freshening-drying phase and reveals stage-specific microbial drivers of quality enhancement. The findings provide theoretical foundations for standardizing CRP aging protocols, directing functional microbial modulation, and constructing quality evaluation systems, while offering methodological insights for processing mechanisms of other dual-purpose medicinal-food resources.

## 2 Materials and methods

### 2.1 Experimental materials

To ensure sample representativeness and homogeneity, fresh citrus fruits (*Citrus reticulata* ‘Dahongpao’) were collected from Zhao Town, Jintang County, Chengdu City, Sichuan Province, China (geographic coordinates: 104.439842°E, 30.856903°N), with consistent origin and cultivar. The fruits were authenticated as mature *C. reticulata* ‘Dahongpao’ by Prof. Yan Zhuyun (College of Pharmacy, Chengdu University of Traditional Chinese Medicine). Fresh pericarps (d0) were manually separated and processed according to traditional methods: sun-dried for 7 days (d7) to obtain the dried intermediate, then stored in woven polypropylene bags under natural aging conditions in a cool, dry, and well-ventilated storage facility at the Chengdu University of Traditional Chinese Medicine (Wenjiang District, Chengdu, China). Sampling spanned the entire 360-day freshening-drying-aging continuum, including fresh pericarp (d0), dried pericarp (d7), and monthly samples from d30 to d360 (14 timepoints in total). During the sampling process, precautions were taken by using sterile gloves to minimize contamination, which aids in maintaining the integrity of the microbial structures present at the time of sampling. All samples were collected in a single batch, and the open sampling protocol is considered acceptable due to the dynamic nature of microbial composition in CRP aging. Samples were analyzed for moisture content, chemical composition, and microbial profiles. Temperature and humidity in the storage environment were monitored using DS1923 data loggers (Shanghai Wodisen Electronics Technology Co., Ltd., China).

### 2.2 Detection of microorganisms in CRP samples with different drying methods

CRP samples were processed using five drying methods: traditional approaches (sun-drying, shade-drying, and oven-drying at 30 °C, 40 °C, or 50 °C) and modern techniques (freeze-drying and vacuum-drying). Post-drying, the CRP surfaces were repeatedly swabbed with sterile cotton swabs. The swab tips were excised for genomic DNA extraction using the cetyltrimethylammonium bromide (CTAB) method. Subsequent steps, including PCR amplification (primer pairs ITS1F/ITS2R), library preparation, sequencing, and bioinformatic analysis, followed the high-throughput sequencing protocols for surface fungi established in our previous study. Sequencing was conducted by Novogene Co., Ltd. (Beijing, China) on the Illumina MiSeq platform (Hu et al., 2025).

### 2.3 Determination of appearance and color value of CRP during the freshening-drying-aging process

During the freshening-drying-aging processing of CRP, the macroscopic characteristics of all samples were documented. Colorimetric parameters were measured using a CM-5 spectrophotometer (Konica Minolta, Japan) under standardized conditions: D65 illuminant, specular component excluded (SCE) reflectance mode, 10° observation angle, and a 30 mm test aperture. The wavelength range was set to 360–740 nm. Prior to sample analysis, the instrument was calibrated with white and black reference tiles. Triplicate measurements were performed for each sample to ensure data reproducibility (Chen, Fu, et al., 2024).

### 2.4 Determination of temperature and humidity, water content and water activity during the process of freshening-drying-aging of CRP

The moisture content of CRP samples was determined according to Method 4 of General Chapter 0832 in Part IV of the Chinese Pharmacopoeia (2020 edition). Water activity was measured using an HD-3 A water activity analyzer (Wuxi Huatec Co., Ltd., China) (Olofsson, 2023). Briefly, samples were pulverized, sieved through a No. 4 mesh (250 μm aperture), and evenly placed in plastic sample dishes. The sensor probe was positioned over the dish, allowed to equilibrate for 5 min until stable readings were achieved, and triplicate measurements were recorded for each sample.

### 2.5 Detection of active substances in the process of freshening-drying-aging of CRP

#### 2.5.1 Total flavonoids and flavonoid components detection

Total flavonoid content in CRP was quantified using an established protocol (Sun et al., 2023), while individual flavonoids (hesperidin, nobiletin, and hesperetin) were analyzed via high-performance liquid chromatography (HPLC) under optimized conditions: a Boston Phlex ODS column (4.6 × 250 mm, 5 μm) with a mobile phase comprising 0.05 % phosphoric acid aqueous solution (A) and acetonitrile (B) in a gradient elution (0–10 min: 15 %–25 % B; 10–25 min: 25 %–35 % B; 25–35 min: 35 %–45 % B; 35–40 min: 45 %–60 % B) at 30 °C and 0.7 mL·min^−1^flow rate. Detection wavelengths were set to 283 nm (hesperidin) and 335 nm (nobiletin, tangeretin) with a 5.0 μL injection volume. For sample preparation, 0.50 g of CRP powder was refluxed with 25 mL methanol at 75 °C for 1 h, cooled, adjusted to initial weight, filtered through a 0.22 μm membrane, and analyzed. Reference solutions included hesperidin (0.543 mg·mL^−1^, nobiletin (0.0413 mg·mL^−1^, and tangeretin (0.0257 mg·mL^−1^, prepared by dissolving standards in methanol and serially diluting to target concentrations (Zheng et al., 2021).

#### 2.5.2 Polysaccharide content determination

Polysaccharide content in CRP was determined using the phenol‑sulfuric acid method (Xu et al., 2025). Briefly, 1.0 g of powdered sample was defatted with 25 mL petroleum ether via reflux at 85 °C for 2 h, filtered, and air-dried. The residue was further refluxed with 30 mL 80 % ethanol at 80 °C for 2 h, filtered, and washed twice with 15 mL 80 % ethanol. The defatted residue was refluxed with 250 mL distilled water for 2.5 h, hot-filtered, and diluted to 250 mL. A glucose standard solution (0.1012 mg/mL) was prepared by dissolving 10.12 mg anhydrous glucose (dried to constant weight at 105 °C) in 100 mL water. Aliquots (0–1.2 mL) of the standard solution were diluted to 2.0 mL with water, mixed with 1.0 mL 5 % phenol and 7.0 mL concentrated sulfuric acid, incubated at 80 °C for 15 min, and cooled to room temperature. Absorbance at 488 nm was measured (blank: water), yielding a linear calibration curve (Y = 0.0493× − 0.019, *R* = 0.9979), where X represents glucose concentration (mg/mL). For sample analysis, 1.0 mL of the CRP extract was processed identically, and polysaccharide content was calculated based on the regression equation.

#### 2.5.3 Volatile oil determination

Weigh approximately 50 g of the sample powder (which has passed through a No. 2 sieve) and transfer it into a 1000 mL round-bottom flask. Add an appropriate volume of distilled water, and then determine the volatile oil content using Method A as outlined in the 2020 edition of the Chinese Pharmacopoeia (General Rule 2204).

#### 2.5.4 Volatile component analysis

Volatile compounds in CRP were analyzed using gas chromatography–mass spectrometry (GC–MS) under the following conditions: an HP-5MS capillary column (5 % phenyl methyl siloxane, 30 m × 0.25 mm × 0.25 μm); inlet temperature 250 °C; helium carrier gas (99.999 %) at 0.8 mL/min; temperature program: initial 40 °C (held for 2 min), ramped at 3 °C/min to 100 °C, then 15 °C/min to 270 °C (held for 2 min); injection volume 1 μL; split ratio 5:1. The mass spectrometer operated in electron ionization (EI) mode (70 eV) with an ion source temperature of 230 °C, scanning *m*/*z* 50–600 in full-scan mode (0.5 s/decade). Total ion chromatograms (TICs) of n-alkane (C7–C30) standards and CRP samples were acquired. Retention indices (RIs) were calculated using the Van Den Dool and Kratz equation: RI = 100(t_x_-t_n_)/(t_n+1_-t_n_). T_x_ is the retention time of the analyzed component, and t_n_ and t_n+1_ are the retention times of the positive alkanes with carbon atoms n and n + 1 respectively, and t_n+1_ > t_x_ > t_n_ (Shi et al., 2024).

### 2.6 Microbial profiling during CRP freshening-drying-aging process

Microbial sampling was conducted to ensure representativeness and universality. Equal aliquots of approximately 30 g of CRP samples were collected from the upper, middle, and lower layers of the woven polypropylene storage bags.Twenty-five intact CRP pieces were randomly selected (zigzag sampling), divided into five subsets (five biological replicates per subset), and processed for microbial analysis as described in Section 2.2.

### 2.7 Data processing

Data were analyzed using SPSS 21.0 statistical software. The results are presented as x ± s. For normally distributed measurement data, one-way analysis of variance (ANOVA) was conducted; for data that do not meet the normality assumption, non-parametric tests were employed.

## 3 Results and discussion

### 3.1 Effects of different drying methods on the microbial community structure of CRP

**Fig. 1:**
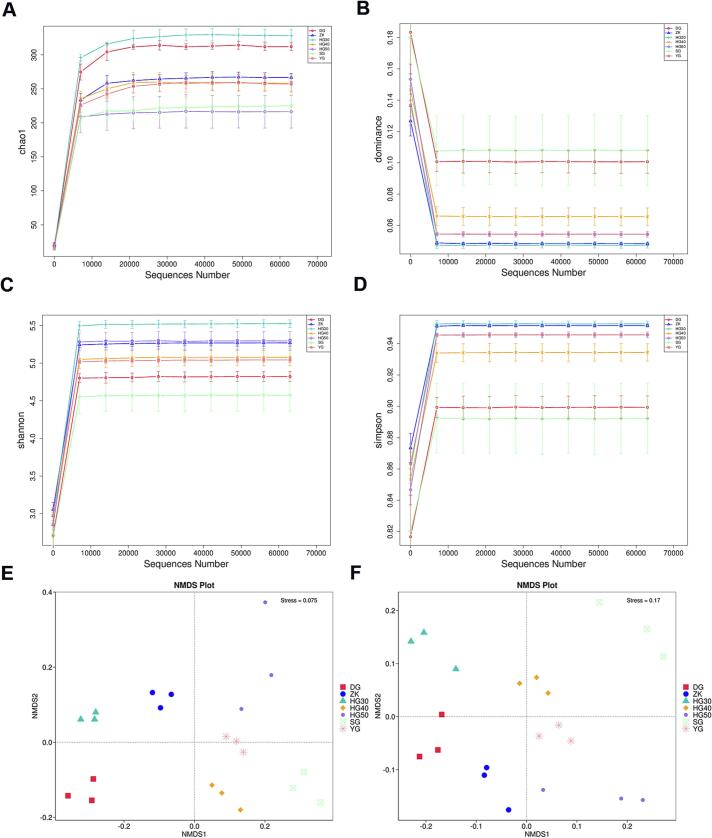
Microbial diversity in CRP samples under different drying methods. (A-B) β diversity analysis; (C—F) α diversity analysis. SG: sun-drying; YG: shade-drying; HG30: oven-drying at 30 °C; HG40: oven-drying at 40 °C; HG50: oven-drying at 50 °C; DG: freeze-drying; ZK: vacuum-drying.

To investigate the effects of drying methods on microbial community structure in CRP, fungal profiles were analyzed across five drying approaches, including oven-drying (30 °C, 40 °C, 50 °C), freeze-drying, vacuum-drying, sun-drying, and shade-drying. Results demonstrated that CRP treated with oven-drying at 30 °C (HG30) exhibited superior microbial *α*-diversity (Fig. 1A-1D). The Chao1 index (573.410) and Shannon index (5.635) were highest in HG30, indicating the richest species richness and most balanced community distribution, respectively. A lower Simpson index (0.953) further confirmed minimal dominance of specific taxa in HG30. *β*-Diversity analysis via NMDS revealed distinct clustering of HG30 samples (coordinates: −0.28 to −0.32/0.06–0.08), suggesting a unique microbial structure (Fig. 1E-1F). Compared to traditional sun-drying (DG) and shade-drying (YG), HG30's mild heating likely promoted proliferation of mesophilic microbes while avoiding thermal inhibition of sensitive taxa observed in high-temperature drying (e.g., HG50). Unlike freeze-drying (ZK) and vacuum-drying (SG), HG30's moderate dehydration rate may foster a microenvironment conducive to multispecies coexistence. This balanced thermo-hygrometric condition likely delayed rapid colonization by dominant taxa, thereby preserving higher diversity. These findings highlight oven-drying at 30 °C as an optimal strategy for balancing microbial diversity and drying efficiency.

Actually, the dynamic relationship between early-stage microbial diversity and late-stage bioactive transformation in CRP aging involves three synergistic mechanisms. First, high initial diversity enhances collaborative metabolism by facilitating interactions among various microbial taxa that engage in mutualistic relationships, thereby releasing polysaccharides and flavonoids as substrates for subsequent functional taxa. (L. Yang et al., 2023). Second, microbial competition under high diversity selectively enriches acid-tolerant and *Xerophilic* functional taxa, establishing stable late-stage consortia. For instance, *Aspergillus* abundance increases from 15 % to 60 % after one year of aging (Melo et al., 2023). Third, diverse enzymatic systems from multispecies communities accelerate glycosidic bond hydrolysis and esterification of flavonoids, thereby enhancing the bioavailability of hesperetin and nobiletin (Shen et al., 2024). These mechanisms underpin our selection of 30 °C oven-drying during the freshening-drying phase (0–7 d) to optimize CRP quality. Experimentally, compared to alternative drying techniques, 30 °C oven-drying yields samples with higher microbial diversity, which is conducive to superior CRP quality development. Furthermore, oven drying offers practical advantages of being readily available, simple to operate, and cost-effective. Synthesizing these considerations, this study adopts 30 °C oven drying as the designated methodology.

### 3.2 Changes of temperature and humidity, water content and water activity during the process of freshening-drying-aging of CRP

**Fig. 2:**
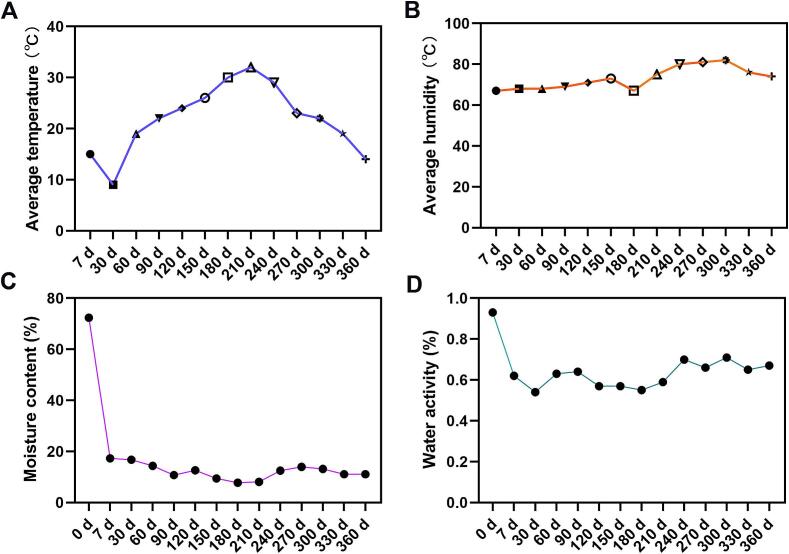
Changes of temperature and humidity (A-B), water content (C) and water activity (D) during the process of freshening-drying-aging of CRP.

The freshening-drying-aging processing of CRP involves dynamic interactions among temperature, humidity, moisture, and water activity, all of which critically influence its final quality. Temperature and humidity data recorded via button-type data loggers revealed fluctuations between 3 °C–36.9 °C and 47 %–89 % relative humidity (RH), respectively (Fig. 2A–2B). High-temperature periods (180–270 d, July to October) and high-humidity phases (270–330 d, October to December) aligned with Chengdu's climatic patterns (hot summers and cold, humid winters), confirming the reliability of environmental monitoring.

Moisture content and water activity exhibited marked variability (Fig. 2C–2D), averaging 12.2 % and 0.62, respectively. During early storage (30–120 d), irregular fluctuations in moisture and water activity suggested contributions from residual physiological metabolism (e.g., enzymatic activity) and microbial adaptation, alongside environmental factors. Mid-storage (180–210 d, July–August) saw the lowest moisture (7.8 %) and water activity (0.55), coinciding with peak summer temperatures (up to 37 °C). Conversely, moisture content peaked at 13 % (Aw = 0.70) by 240 d (September–October), driven by elevated humidity (RH = 81 %). These findings underscore the necessity for vigilant quality control during high-risk periods (August–October), including regular inspection for mold and insect infestation, as well as timely sun-redrying to prevent spoilage—a practice consistent with traditional CRP aging protocols.

### 3.3 Changes in appearance of CRP during the process of freshening-drying-aging

**Fig. 3:**
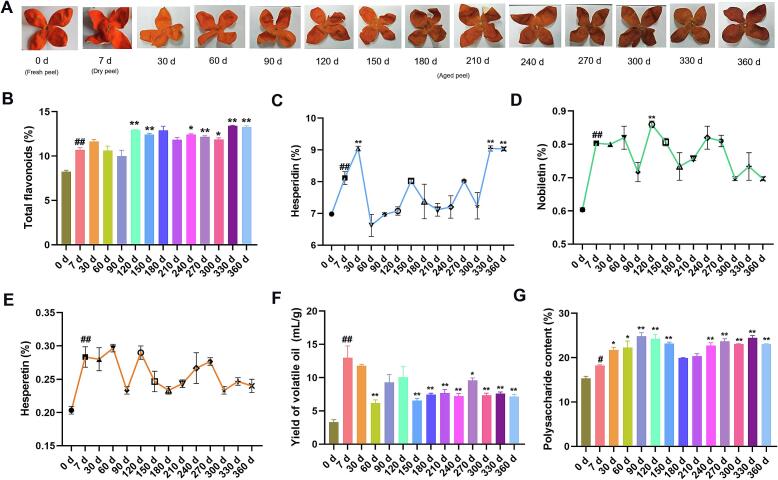
Changes in the characteristics and active ingredient content of CRP during the freshening-drying-aging process. (A) Changes in characteristics; (B) Variation in total flavonoid content; (C) Yield of volatile oil; (D) Content of polysaccharides; (*E*-F) Content of hesperidin, naringin, and naringenin.

During the freshening-drying-aging processing of CRP, significant changes in macroscopic characteristics were observed (Fig. 3A). The pericarp color transitioned from initial bright orange-red to reddish-brown, yellowish-brown, and finally dark brown. The aroma evolved from intensely pungent to mellow and earthy, while texture shifted from brittle (0–240 d) to slightly softened (270–300 d), correlating with temperature and humidity fluctuations in the storage environment. Notably, elevated temperatures typically accelerate moisture loss, leading to structural hardening, though this trend was modulated by humidity variations in later stages.

Colorimetric analysis (Table 1) revealed dynamic trends: L* values (lightness) initially increased (0–180 d) before declining, indicating gradual darkening. Similarly, a* values (red-green axis) decreased overall, while b* values (yellow-blue axis) peaked at 180 d and subsequently declined, aligning with visual observations of progressive darkening. These shifts are consistent with the color specifications for CRP in the Chinese Pharmacopoeia (2020 edition), which mandates prolonged aging for quality authentication.

**Table 1:** Color value of CRP during freshening-drying-aging process (*n* = 3).

Group	L*(brightness)	a*(Red green degree)	*b*^⁎^(Blue yellow degree)
0	57.36 ± 0.09	16.19 ± 0.02	31.17 ± 0.01
7d	67.69 ± 0.40	18.04 ± 0.18	39.11 ± 0.56
30d	70.08 ± 0.25	20.17 ± 0.15	44.19 ± 0.19
60d	70.42 ± 0.33	18.43 ± 0.14	47.28 ± 0.40
90d	71.51 ± 0.06	17.76 ± 0.10	48.60 ± 0.22
120d	70.28 ± 0.09	16.31 ± 0.09	45.04 ± 0.11
150d	72.61 ± 0.21	13.92 ± 0.20	40.69 ± 0.30
180d	72.14 ± 0.13	13.19 ± 0.05*	36.74 ± 0.16
210d	71.77 ± 0.05	12.08 ± 0.11	35.07 ± 0.10
240d	71.20 ± 0.57	10.95 ± 0.07	33.72 ± 0.29
270d	71.43 ± 0.51	10.15 ± 0.04	33.52 ± 0.31
300d	71.54 ± 0.21	10.57 ± 0.11	33.39 ± 0.08
330d	69.77 ± 0.34	9.74 ± 0.02	33.56 ± 0.43
360d	69.93 ± 0.07	9.80 ± 0.04	33.10 ± 0.29

Notably, CRP's phenotypic evolution is closely linked to microbial activity. Our prior studies demonstrated that microbial communities drive both structural degradation and pigment biosynthesis (F. Wang et al., 2023). Reported evidence further highlights microbial roles in fermentative processes that enhance medicinal efficacy and flavor complexity (Gao et al., 2023). In food science, color dynamics often serve as critical indicators of quality and safety, with microbial metabolism being a key modulator. These findings provide foundational insights into the interplay between microbiota, bioactive components, and flavor compounds during CRP aging, as explored in subsequent sections.

### 3.4 Changes in total flavonoids and specific flavonoid compounds during the process of freshening-drying-aging of CRP

Flavonoids are the main active compounds in CRP, and their content and proportions directly influence its pharmacological effects, such as antioxidant and anti-inflammatory activities (Zhang et al., 2022). Studying the changes in flavonoid content during the freshening-drying-aging process of CRP is vital for understanding its quality variations. The results indicate a general increasing trend in flavonoid content throughout the freshening-drying-aging process. Compared to the fresh peel sample at 0 days, the total flavonoid content significantly increased in the samples dried for 7 days (*P* < 0.01) (Fig. 3B). When comparing the 7-day dried samples with those stored for 120, 150, 240, 270, 300, 330, and 360 days, significant differences in total flavonoid content were observed (*P* < 0.01). The 7-day samples showed a total flavonoid content of 10.4 %, while the 330-day and 360-day samples displayed values of 13.4 % and 13.2 %, respectively. Notably, the total flavonoid content decreased slightly from 330 days to 360 days, but both the 330-day and 360-day samples showed a significant increase of approximately 3 % compared to the 7-day samples (*P* < 0.01). The content of hesperidin exhibited substantial fluctuations throughout the process, generally showing an upward trend (Fig. 3C). Compared to the fresh peel sample at 0 days, the hesperidin content significantly increased in the samples dried for 7 days (*P* < 0.01). Relative to the 7-day dried samples, the hesperidin content increased in the 30-day samples, but sharply decreased from 30 to 60 days, followed by a gradual increase. The average hesperidin content across all storage periods ranged from 7 % to 8 %, with significant differences noted in the 330-day and 360-day samples when compared to the 7-day sample (*P* < 0.01). Specifically, the hesperidin content was 8.15 % for the 7-day sample, while the 330-day and 360-day samples had contents of 9.07 % and 9.08 %, respectively. The contents of nobiletin and hesperetin showed significant increases from the fresh to the dried samples (*P* < 0.01), with nobiletin content rising from 0.61 % to 0.81 % and tangeretin content increasing from 0.21 % to 0.28 %. During the storage period from 7 to 360 days, the levels of both compounds remained relatively stable, fluctuating between 0.72 and 0.84 % and 0.24–0.28 %, respectively. (Figs. 3D-3E). The metabolic footprints of the flavonoid components nobiletin, hesperidin, and hesperetin demonstrate similar patterns of content variation, as shown in Fig. 5A. Metabolic footprint refers to the specific profiles of metabolites and their transformations within biological systems, reflecting the underlying biochemical pathways. For instance, hesperidin, a glycoside, can be hydrolyzed to yield hesperetin, its aglycone form. This interrelationship highlights the metabolic conversions among these compounds, emphasizing their interconnectedness and potential implications for biological activity and health benefits.

During the freshening-drying-aging process of CRP, the content of flavonoids gradually increases, which is consistent with previously reported findings (He et al., 2021). Current research generally suggests that various enzymatic reactions and redox processes within CRP promote the synthesis of flavonoids (Guo et al., 2021). Additionally, microbial metabolic activities during storage may also positively influence the abundance of flavonoid compounds. However, some studies indicate that the changes in flavonoid content are not unidirectional; fluctuations in storage temperature and humidity or prolonged storage times can lead to the degradation of flavonoids, which aligns with the fluctuating patterns observed in this study (Tao et al., 2022). In fact, significant changes in flavonoid content over extended storage times are beneficial for enhancing the quality of CRP. Flavonoids in CRP, primarily including hesperidin and naringin, exhibit multiple beneficial effects, such as antioxidant, anti-inflammatory, and immune-enhancing properties (Q. Yu et al., 2022). As CRP ages, the increase in flavonoid content correlates with improved pharmacological activities, thereby not only enhancing the quality of CRP but also validating the traditional belief that “the longer it is aged, the better it becomes” (Qin, Li, et al., 2024; Qin, Zhao, et al., 2024).

### 3.5 Changes of volatile oil yield and volatile components during the process of freshening-drying-aging of CRP

**Fig. 4:**
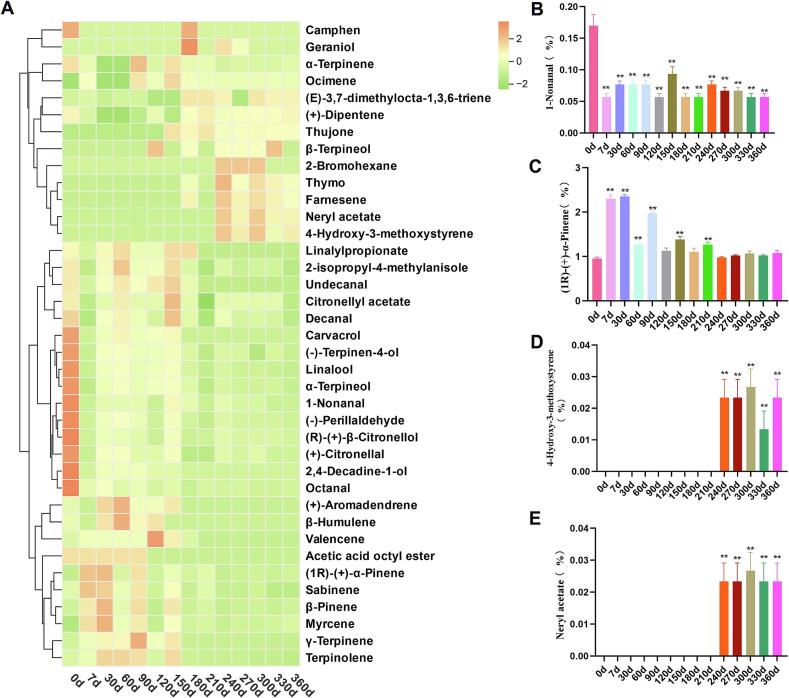
Changes in volatile components during the freshening-drying-aging process of CRP. (A) Component heatmap; (B-E) Relative content of representative compounds (Z)-3,7-dimethyl-2,6-octadien-1-ol acetate, 4-vinyl-2-methoxyphenol, nonanal, and pinene.

**Fig. 5:**
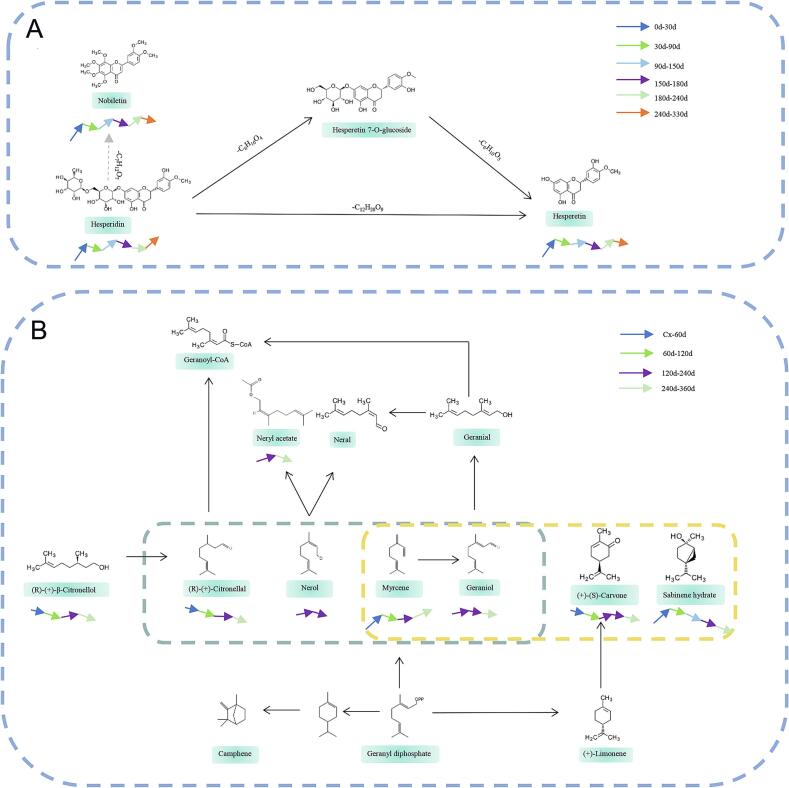
The conversion relationships of three flavonoid compounds and the metabolic footprints of volatile components.(A) The conversion relationships among hesperidin, nobiletin, and tangeretin; (B) Metabolic footprints of representative flavor compounds.

Volatile oils, critical determinants of CRP's sensory and bioactive quality, exhibited a declining trend during the freshening-drying-aging continuum. The yield decreased significantly from 14 % in dried samples (7 d) to 6 % by 360 d (*P* < 0.05), with the most pronounced reduction occurring within the first 60 d (Fig. 3F). This decline underscores the volatility-driven loss of aromatic compounds, suggesting dried samples (7 days) as a preferable source for essential oil extraction. GC–MS analysis identified 85 volatile compounds (Table 2), with the highest diversity (56 compounds) observed at 270 d. Six constituents exceeded 1 % relative abundance: limonene, pinene, myrcene, *γ*-terpinene, linalool, and carvacrol. Notable compositional shifts occurred post-150 d, marked by the emergence of new compounds (e.g., thujone, geraniol, thymol, nerol acetate, (−)-*α*-cubebene, α-farnesene) and the disappearance of others (e.g., *p*-cymene, verbenone, carvone, carvacrol, linalyl propionate, aromadendrene, valencene, elemol, *β*-humulene). Heatmap analysis (Fig. 4A) categorized volatile dynamics into three clusters: (I) steadily decreasing (e.g., nonanal, reducing greasy/stimulant odors; Fig. 4B), (II) initial increase followed by decline (e.g., pinene, mitigating pungency by 240–360 d; Fig. 4C), and (III) progressively increasing (e.g., (Z)-3,7-dimethyl-2,6-octadien-1-ol acetate and 4-vinyl-2-methoxyphenol, imparting floral notes; Fig. 4D–4E). These trends, particularly the late-stage emergence of unique compounds (150–360 d), highlight dynamic chemical remodeling during CRP aging. The metabolic footprints of these representative volatile components clearly reveal the patterns of content variation (Fig. 5B). In particular, the content of (*R*)-(+)-*β*-Citronellol decreases continuously with the aging process. The compounds nerol and neryl acetate only appear in the later stages of aging. It is inferred that there may be a close transformation relationship among these three compounds.

**Table 2:** Volatile components of CRP during freshening-drying-aging process.

No.	RI	RI*	Name	Relative content(%)													
				CX	7d	30d	60d	90d	120d	150d	180d	210d	240d	270d	300d	330d	360d
**1**	920	928	Thujone, (A + B)(SG)	–	–	–	–	–	–	0.266	0.22	0.26	0.18	0.20	0.21	0.20	0.20
**2**	930	930	(1*R*)-(+)-α-Pinene	0.93	2.24	2.33	1.20	1.95	1.09	1.42	1.15	1.30	0.96	1.03	1.10	1.03	1.11
3	943	943	Camphen	0.021	0.02	0.02	0.02	0.02	0.02	0.02	0.021	0.02	0.02	0.02	0.02	0.02	0.02
4	946	941	2-Bromohexane	–	–	–	–	–	–	–	–	–	0.01	0.01	0.01	–	–
**5**	968	975	Sabinene	0.12	0.20	0.189	0.13	0.17	0.10	0.15	0.092	0.12	0.09	0.09	0.10	0.08	0.08
**6**	970	970	*β*-Pinene	0.47	0.76	0.94	0.58	0.80	0.42	0.77	0.464	0.51	0.43	0.45	0.47	0.44	0.48
**7**	986	981	Myrcene	1.62	2.73	3.12	2.26	2.60	1.82	2.42	1.958	2.01	1.84	1.94	1.92	1.96	2.06
**8**	998	1005	Octanal	0.99	0.27	0.39	0.24	0.30	0.17	0.24	0.102	0.18	0.15	0.16	0.14	0.14	0.15
9	1011	1008	*α*-Terpinene	0.22	0.16	0.07	0.07	0.26	0.14	0.22	0.158	0.16	0.13	0.14	0.14	0.14	0.15
10	1020	1011	4-Isopropyltoluene	–	0.03	–	–	–	–	–	–	–	–	–	–	–	–
11	1019	1013	M-Cymene	–	–	0.09	0.05	–	–	–	–	–	–	–	–	–	–
**12**	1032	1020	(+)-Dipentene	87.09	81.68	75.08	75.01	78.19	83.33	77.54	85.97	87.71	85.21	86.22	85.83	86.20	87.06
13	1037	1036	(E)-3,7-dimethylocta-1,3,6-triene	0.03	0.04	0.02	0.03	0.03	–	–	0.09	0.09	0.08	–	0.09	0.08	0.08
14	1047	1041	Ocimene	–	0.15	–	–	0.19	0.13	0.21	0.14	0.13	0.12	0.12	0.12	0.13	0.12
15	1044	1044	3,5,5-trimethylcyclohex-3-en-1-one	0.10	–	0.01	–	–	–	–	–	–	–	–	–	–	–
**16**	1057	1053	*γ*-Terpinene	5.65	6.99	8.00	7.92	10.42	7.12	8.40	5.30	5.29	5.35	5.32	5.31	5.23	5.22
17	1070	1228	Geraniol	–	–	–	–	–	–	–	0.04	–	0.02	0.01	–	–	–
**18**	1087	1080	Terpinolene	0.35	0.39	0.52	0.52	0.50	0.34	0.51	0.31	0.30	0.30	0.30	0.29	0.29	0.28
**19**	1102	1092	Linalool	6.97	0.96	2.89	3.08	2.46	2.76	3.30	1.54	0.79	1.61	1.17	1.27	1.02	0.84
**20**	1106	1104	1-Nonanal	0.16	0.06	0.08	0.08	0.08	0.06	0.10	0.0.08	0.06	0.08	0.07	0.07	0.06	0.06
21	1139	1221	[1S,2R,6R,7R,8S,(+)]-1,3-Dimethyl-8-(1-methylethyl)tricyclo[4.4.0.02,7]deca-3-ene	–	–	–	–	–	–	0.01	0.02	–	–	–	–	–	–
22	1141	1139	(+)-Trans-L 1,2-epoxide	0.02	–	–	–	–	–	–	–	–	–	–	–	–	–
23	1113	1119	2-Ethyl-*p*-xylene	–	–	–	–	–	–	0.01	–	–	–	–	–	–	–
24	1124	1123	trans−4-(isopropyl)-1-methylcyclohex-2-en-1-ol	–	–	–	–	–	–	0.02	0.01	–	–	–	–	–	–
25	1143	1134	(3E,5E)-2,6-Dimethyl-1,3,5,7-octatetrene	–	–	–	–	–	–	0.03	0.01	–	–	–	–	–	–
26	1149	1152	Isopulegol	–	–	–	–	–	–	0.02	–	–	–	–	–	–	–
**27**	1151	1158	*β*-Terpineol	–	–	–	–	–	0.02	–	0.01	–	0.01	0.01	0.01	0.02	–
28	1158	1152	(+)-Citronellal	0.24	0.02	0.10	0.10	0.06	0.06	0.11	–	–	0.06	0.04	0.06	0.03	0.03
29	1182	1175	(−)-Terpinen-4-ol	0.31	0.04	0.14	0.14	0.12	0.12	0.13	0.08	0.02	0.08	0.07	–	0.07	0.04
**30**	1193	1192	*α*-Terpineol	0.79	0.07	0.31	0.38	0.28	0.34	0.35	0.23	0.06	0.20	0.15	0.16	0.17	0.11
31	1197	1199	-4-Undecenal, (4E)-	–	–	–	–	–	–	0.01	–	–		–	–	–	–
32	1203	1271	2-Ethoxybenzaldehyde	–	–	–	–	–	–	–	–	–	0.01	–	–	–	–
33	1211	1204	Decanal	0.28	0.11	0.22	0.24	0.20	0.16	0.30	0.18	0.10	0.20	0.16	0.17	0.16	0.12
34	1217	1191	Acetic acid octyl ester	0.01	0.01	0.01	0.01	0.01	–	–	–	–		–	–	–	–
35	1218	1243	2-Acetyl-3,5-dimethylpyrazine	–	–	–	–	–	–	–	–	–	0.02	–	0.01	–	–
36	1225	1228	Verbenone	0.03	–	–	0.02	0.01	0.02	0.03	0.01	–		–	–	–	–
37	1236	1220	(*R*)-(+)-*β*-Citronellol	0.38	0.01	0.09	0.08	0.06	–	0.11	–	0.01	0.06	0.04	0.04	0.04	0.02
38	1242	1244	2-isopropyl-4-methylanisole	0.34	0.15	0.31	0.41	0.27	0.28	0.37	0.22	0.11	0.22	0.17	0.19	0.19	0.13
39	1249	1240	Citral	0.06	–	0.02	0.02	0.01	–	–	–	–	0.01	–	–	–	–
40	1253	1246	Carvone	0.03	–	–	0.02	0.01	0.01	0.02	–	–	0.01	–	–	–	–
41	1248	1233	Heptanenitrile, 4-acetyl-	–	–	–	–	–	–	–	–	–	0.01	–	0.01	–	–
42	1254	1250	trans-Citral trans-3,7-Dimethyl-octa-2,6-dien-1-al	–	–	–	–	–	–	–	–	–	0.02	–	–	–	–
**43**	1262	1090	Thymol	–	–	–	–	–	–	–	0.12	0.02	0.33	0.16	0.24	0.19	0.17
44	1264	1228	Nerol	–	–	–	–	–	–	0.02	–	–	–	–	–	–	–
45	1270	1240	3-Heptylacrolein	–	–	–	–	–	–	0.03	–	–	–	–	–	–	–
46	1268	1273	2,4-Decadine-1-ol	0.09	–	0.02	0.02	0.02	0.01	0.01	–	–	–	–	–	–	–
47	1280	1276	(−)-Perillaldehyde	0.30	0.02	0.09	0.11	0.08	0.02	0.10	0.03	–	0.05	0.03	0.03	0.03	0.02
48	1296	1297	Carvacrol	1.05	0.03	0.33	0.50	0.32	0.37	0.47	–	–	–	–	–	–	–
**49**	1281	1272	4-Hydroxy-3-methoxystyrene	–	–	–	–	–	–	–	–	–	0.03	0.02	0.03	0.01	0.02
**50**	1306	1342	Neryl acetate	–	–	–	–	–	–	–	–	–	0.03	0.02	0.03	0.02	0.02
**51**	1309	1303	Undecanal	0.04	0.01	0.03	0.04	0.03	0.04	0.05	0.02	–	–	–	–	–	–
52	1323	1319	trans,trans-2,4-undecadienal	–	–	–	–	–	–	0.02	–	–	–	–	–	–	–
53	1344	1334	*δ*-elemene,(3R-trans)-4-ethenyl-4-methyl-3-(1-methylethenyl)-1-(1-methylethyl)-cyclohexene,(1*S*,2*R*)-(−)-2-isopropenyl-1-vinyl-p-menth-3-ene	0.07	0.03	0.12	0.18	0.09	0.10	0.19	0.09	0.02	0.13	0.08	0.11	0.09	0.07
54	1358	1331	Citronellyl acetate	0.03	0.01	0.02	0.03	0.02	0.02	0.04	0.02	–	0.02	0.02	0.02	0.02	0.01
**55**	1369	1371	Linalylpropionate	0.03	0.01	0.03	0.04	0.02	0.02	0.04	0.04	–	–	–	–	–	–
56	1335	1339	cubebene	–	–	–	–	–	–	–	–	–	0.03	0.02	0.02	0.02	0.01
57	1386	1376	(−)-*α*-Copaene	0.01	0.01	0.02	–	–	–	0.02	0.01	–	–	–	–	–	–
58	1313	1344	(−)-*α*-Cubebene	–	–	–	–	–	–	–	–	–	0.02	0.01	0.01	0.01	–
59	1387	1360	Geranyl acetate	0.02	–	0.02	0.02	0.01	0.01	0.03	0.01	–	0.02	0.02	0.02	0.02	–
60	1394	1396	7-Tetradecene	–	–	–	–	–	–	0.01	–	–	–	–	–	–	–
61	1398	1398	*β*-Elemene	0.03	0.02	0.06	0.08	0.04	0.01	0.10	0.05	–	0.07	0.05	0.06	0.05	0.04
62	1411	1402	Dodecyl aldehyde	0.02	–	0.04	0.04	0.03	0.04	0.06	0.03	–	0.04	–	0.03	0.02	0.02
**63**	1417	1421	Dihydro cuminyl acetate	0.03	0.03	0.03	0.03	0.03	0.03	0.03	–	–	–	–	–	–	–
64	1434	1424	*β*-Caryophyllene	–	–	–	–	–	–	0.02	–	–	–	–	–	–	–
**65**	1443	1439	(+)-Aromadendrene	0.02	–	0.06	0.08	0.03	0.03	0.05	–	–	–	–	–	–	–
66	1468	1456	*α*-Caryophyllene	0.01	0.01	0.02	0.03	0.02	0.01	0.04	0.02	–	0.04	0.02	0.02	0.02	0.02
67	1497	1501	germacreneD,1-methyl-5-methylene-8-(1-methylethyl)-1,6-cyclodecadiene	–	–	–	–	–	–	0.18	0.04	–	0.09	0.05	0.06	0.05	0.06
68	1512	1511	1-methyl-4-(1-methylethylidene)-2-(1-methylvinyl)-1-vinylcyclohexane	0.02	0.01	0.08	0.02	0.04	0.08	0.13	0.11	0.01	0.18	0.11	0.15	0.12	0.10
69	1163	1132	1,3,5-Triisopropylbenzene	–	–	–	–	–		–	–	–		–	–		–
70	1166	1432	(−)-*γ*-elemene,1-ethenyl-1-methyl-2-(1-methylethenyl)-4-(1-methylethylidene)-cyclohexane,*γ*-elemene	–	–	–	–	–	–	–	0.01	–		–	–	–	–
**71**	1167	1458	Farnesene	–	–	–	–	–	–	–	0.04	–	0.06	0.04	0.05	0.04	0.03
72	1367	1447	tetradecamethylcycloheptasiloxane	–	–	–	–	–	–	–	–	–	0.03	–	–	0.01	0.03
73	1370	1431	1-methyl-4-(1-methylethylidene)-2-(1-methylvinyl)-1-vinylcyclohexane	0.02	0.01	0.08	0.02	0.04	0.08	0.13	–	–	0.01	–	0.01	–	–
74	1374	1431	[S,(−)]-2,3,4,4a,5,6-Hexahydro-1,4a-dimethyl-7-(1-methylethyl)naphthalene	–	–	–	–	–	–	–	–	–	0.01	–	–	–	–
75	1432	1598	(2*R*)-2,3,4,4a,5,6,7,8-Octahydro-α,α,4a*β*,8*β*-tetramethyl-2-naphthalenemethanol	–	–	–	–	–	–	–	–	–	0.04	0.02	0.03	0.02	0.03
76	1466	1586	2-pentylnon-2-enal	–	–	–	–	–	–	–	–	–	0.02	–	0.02	0.01	0.01
77	1494	1500	1-Nitroadamantane	–	–	0.01	–	–	–	–	–	–	0.01	–	–	–	–
78	1520	1515	Valencene	0.01	0.02	0.02	0.02	0.02	0.06	0.03	0.01	–	–	–	–	–	–
79	1526	1510	*α*-Bulnesene	–	–	–	–	–	0.01	–	–	–	–	–	–	–	–
80	1536	1537	(1*S*,2*S*,4*R*)-(−)-alpha,alpha-d	0.02	–	0.04	0.05	0.03	0.03	0.06	0.03	–	0.03	0.02	0.03	0.03	0.02
81	1553	1553	imethyl-1-vinyl-o-menth-8-ene-4-methanol	0.01	–	0.01	0.01	0.01	–	–	–	–	–	–	–	–	–
82	1564	1574	Nerolidol	–	–	–	–	–	–	0.03	–	–	–	–	–	–	–
**83**	1578	1574	*β*-Humulene	0.02	–	0.05	0.08	0.04	0.05	–	–	–	–	–	–	–	–
84	1653	1646	Cedren-13-ol, 8-	–	–	–	–	–	–	0.02	–	–	–	–	–	–	–
85	1679	1644	*β*-Eudesmol	–	–	–	–	–	–	0.03	–	–	–	–	–	–	–

Note: “-” mean not detected; “RI” is the measured value; “Ri*” is the retrieved value of NIST11.

The complexity of volatile evolution in CRP aligns with prior studies emphasizing the impacts of drying, storage duration, and environmental conditions (Li, Zhu, et al., 2022). While traditional views associate aging with enhanced aroma through oxidative and enzymatic transformations, our findings reveal a dual process of degradation and synthesis. The late-stage emergence of floral terpenoids and phenolic derivatives (e.g., 4-vinyl-2-methoxyphenol) suggests microbial or abiotic catalysis, a hypothesis unexplored in existing literature. Notably, the decline in aldehydes (e.g., nonanal) and stabilization of pinene levels correlate with sensory refinement, supporting the empirical preference for aged CRP. However, the mechanistic role of microbiota in these transformations remains uncharacterized, warranting further investigation into microbial-volatile interactions to optimize CRP's aromatic and therapeutic profiles.

### 3.6 Changes of microorganisms in the process of freshening-drying-aging of CRP

**Supplementary Figure S1:**
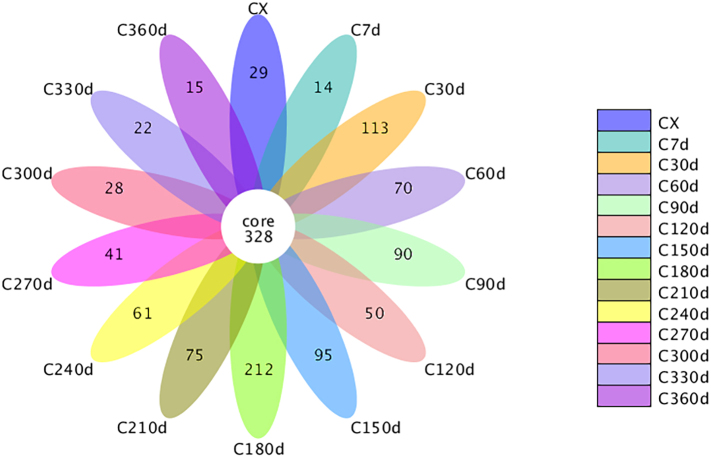
**Figure S1 Petal diagram.** Each petal in the petal diagram represents one (group) of samples, different colours represent different samples (groups), the CORE numbers in the middle represent the number of OTUs common to all samples, and the numbers on the petals represent the number of OTUs specific to that sample (group).

**Supplementary Figure S2:**
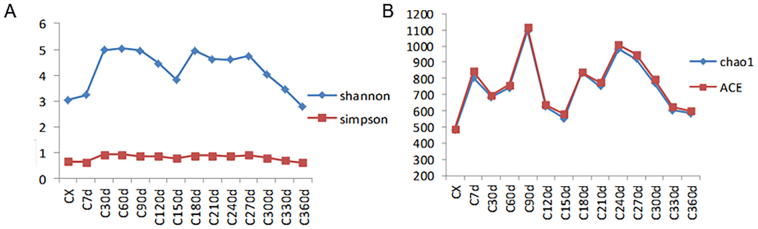
**Figure S2 Plot of changes in diversity index for each group of samples.** (A) The index of Shannon and Simpson; (B) The index of Chao1and ACE.

Following sequencing on the Illumina HiSeq platform, raw paired-end (PE) reads were processed through quality control and chimera filtering to generate clean tags and effective tags, respectively. Operational Taxonomic Units (OTUs) were clustered at 97 % identity threshold using effective tags. Taxonomic annotation of representative OTU sequences revealed 328 shared OTUs across all storage periods (Fig. S1), with 113 and 212 unique OTUs specifically identified at 30 d and 180 d, respectively. Alpha diversity indices (Ace and Chao for community richness; Shannon and Simpson for diversity) showed significant increases at 90 d (Fig. S2), coinciding with spring conditions characterized by elevated temperature and humidity that promoted microbial proliferation. Notably, these indices declined by 270 d during early winter in Chengdu, when reduced temperatures suppressed microbial metabolic activities. These results demonstrate abundant active microorganisms on CRP surfaces during the freshening-drying transition, though desiccation progressively inhibited microbial activity. Previous studies confirming the persistence of xerotolerant microorganisms in dried CRP align with these findings (Liu et al., 2025).

**Fig. 6:**
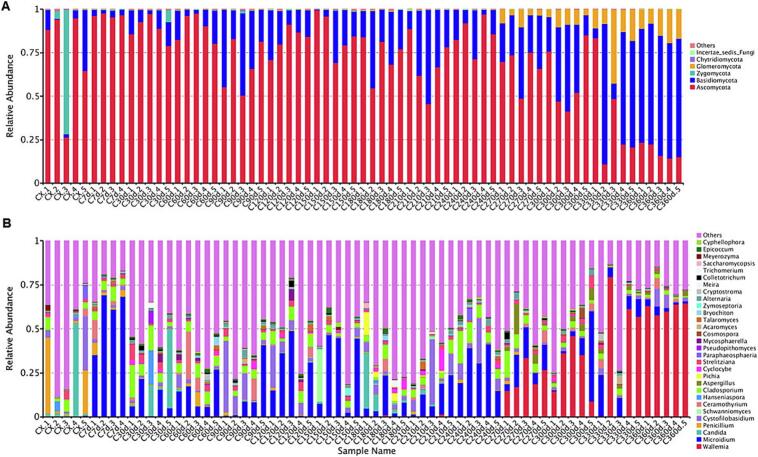
Changes in the abundance of fungi during the process of freshening-drying-aging of CRP. (A) Phylum level; (B) genus level.

Fungal community composition analysis at different storage stages revealed five phyla: *Ascomycota* (65 %), *Basidiomycota* (34 %), with minor contributions from *Zygomycota*, *Chytridiomycota*, and *Glomeromycota* (Fig. 6A). Seventeen genera were identified, dominated by *Microidium* (35.37 %), *Wallemia* (22.75 %), *Cladosporium* (9.41 %), *Candida* (5.19 %), *Ceramothyrium* (3.56 %), *Penicillium* (3.52 %), and *Strelitziana* (2.87 %), while *Aspergillus* (1.36 %) and other genera accounted for <1 % (Fig. 6B). Dynamic shifts were observed: *Ascomycota* abundance decreased progressively, whereas *Basidiomycota* increased during storage. *Glomeromycota* emerged at 270 d with gradual enrichment, while *Zygomycota* was exclusively detected in fresh samples. *Penicillium* dominated fresh samples but declined post-drying, whereas *Aspergillus* appeared only during late storage (270–360 d). *Penicillium* digitatum was primarily distributed in fresh and dried samples.

**Supplementary Figure S3:**
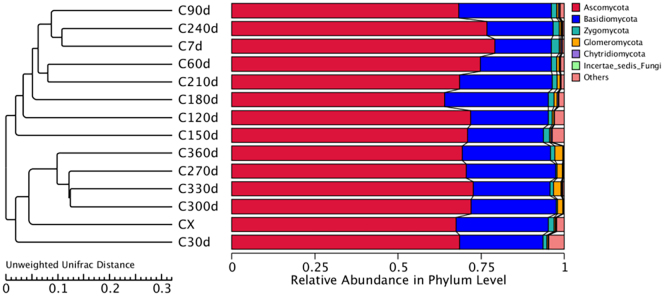
Figure S3 UPGMA clustering tree based on Weighted Unifrac distance.

Dominant genera (Z > 1) included *Penicillium* in fresh samples and late-emerging *Aspergillus* (270–360 d) (Table 3), consistent with community composition trends. UPGMA clustering divided CRP samples into three distinct groups: fresh (0 d) and dried (30 d) samples formed separate clusters; mid-storage samples (60–240 d) clustered together; late-storage samples (270–360 d) formed the third cluster (Fig. S3). Differential species analysis identified maximum unique taxa in fresh samples (0 d), with comparable numbers in dried/stored samples. Crucially, *Aspergillus* emerged as both dominant and differential genus during late storage (270–360 d), absent in earlier stages (0–240 d).

**Table 3:** Distribution of dominant bacterial genera in the freshening-drying-aging process of CRP.

No.	Dominant fungi
CX(C0d)	*Penicillium, Meyerozyma, Paraphaeosphaeriae, Candida*
C7d	*Microidium*
C30d	*Strelitziana, Hanseniaspora, Aureobasidium, Trichomerium, Pseudopithomyces, Candida, Colletotrichum, Cladosporium*
C60d	*Kockovaella, Cryptostroma, Ceramothyrium, Epicoccum, Bryochiton*
C90d	*Cryptococcus, Kockovaella, Cystofilobasidium, Alternaria*
C120d	*Colletotrichum, Mycosphaerella, Talaromyces*
C150d	*Zymoseptoria, Cyphellophora, Schwanniomyces*
C180d	*Kockovaella, Pichia, Meira, Saccharomycopsis, Erythrobasidium, Cyclocybe*
C210d	*Erythrobasidium, Cyclocybe, Talaromyces*
C240d	*Paraphaeosphaeria, Alternaria, Khuskia, Epicoccum, Cladosporium*
C270d	***Aspergillus**, Colletotrichum, Khuskia；*
C300d	*Cosmospora, **Aspergillus***
C330d	*Acaromyces, Cryptostroma, **Aspergillus***
C360d	*Wallemia*

Existing research suggests microbial community shifts during CRP aging correlate with enhanced aroma profiles and modified bioactive components, mediated through microbial diversity and metabolic transformations (Yang et al., 2022). The temporal emergence of dominant genera identified in this study likely drives quality alterations in CRP. These findings provide critical scientific insights for optimizing processing and storage protocols, emphasizing the functional significance of stage-specific microbial consortia in shaping CRP's medicinal properties.

### 3.7 Correlation analysis between chemical components and microorganisms in the process of freshening-drying-aging of CRP

**Fig. 7:**
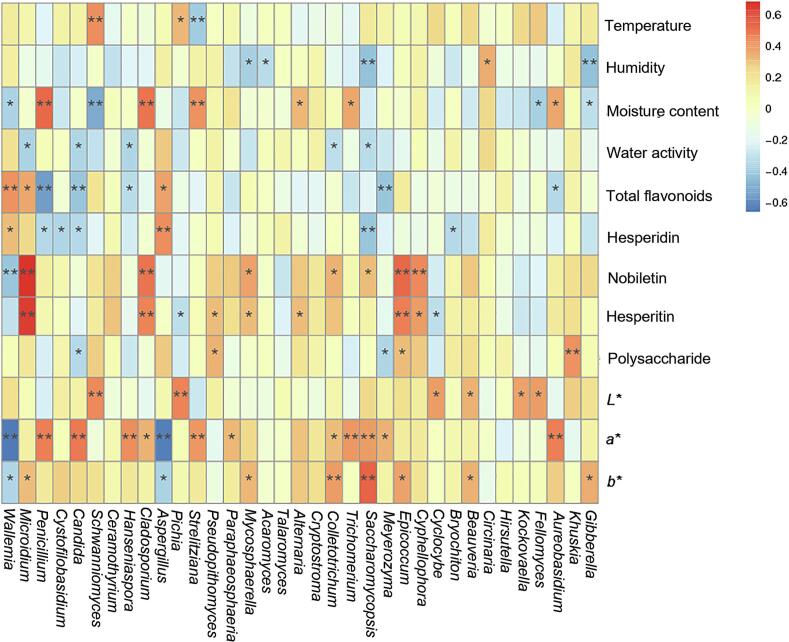
Correlation analysis of active ingredients and microorganisms in the process of freshening-drying-aging of CRP.

**Supplementary Figure S4:**
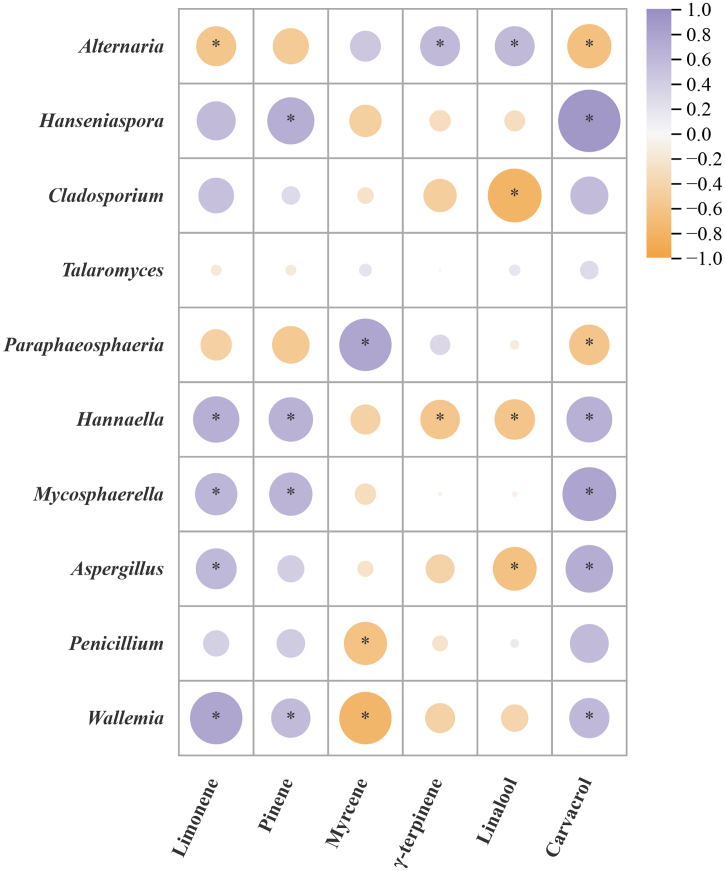
Figure S4 Correlation analysis of main volatile components and microorganisms in the process of freshening-drying-aging of CRP

To investigate the intrinsic relationship between chemical components and microbial communities during the freshening-drying-aging process of CRP, this study conducted correlation analyses between chemical constituents and microbial dynamics (Fig. 7). The results revealed that environmental factors, including temperature, humidity, water activity (Aw), and moisture content, significantly influenced microbial abundance. For instance, *Wallemia* exhibited a negative correlation with moisture content (*p* < 0.05), while temperature showed a positive correlation with *Schwanniomyces*, and humidity was negatively correlated with *Gibberella* (*p* < 0.05). Conversely, microbial shifts impacted chemical composition: *Wallemia* and *Aspergillus* demonstrated positive correlations with total flavonoids and hesperidin content *(p* < 0.05), whereas *Penicillium*, *Candida*, and *Aureobasidium* were negatively correlated with these components (*p* < 0.05). *Aspergillus* is positively correlated with limonene and carvacrol, and negatively correlated with linalool. Other genera, such as *Hanseniaspora* and *Cladosporium*, can affect volatile components, while *Talaromyces* is an exception (Fig. S4).Our prior chemical analyses indicated that fresh CRP contains high volatile oil levels, which gradually decline during drying and aging, while total flavonoids and hesperidin increase significantly (Wang et al., 2023; Yang et al., 2022). This aligns with the current correlation results. The reduction in volatile oil is attributed to its inherent volatility, whereas the 40–60 % increase in total flavonoids corresponds to microbial secondary metabolite accumulation. Notably, *Aspergillus* not only influences CRP's flavor and aroma but also enhances flavonoid accumulation via metabolic activities, underscoring its pivotal role in biotransformation during aging. Consistent with earlier microbial analyses, *Aspergillus* was absent during early storage (0–240 d) but emerged as a dominant and differential genus in later stages (270–360 d). These findings suggest that the aging period from 270 to 360 days represents a critical phase for CRP quality formation.

## 4 Conclusion

Citri reticulatae pericarpium (CRP) develops its distinctive quality through sequential phases of fresh material preparation, drying, and aging. This study systematically elucidates the dynamic evolution of bioactive components and flavor metabolites across the entire freshening-drying-aging continuum of CRP. The results confirm that drying methods exert a preconditioning effect on subsequent aging by modulating microbial community structures. Specifically, drying at 30 °C optimally preserved the diversity of dominant microbial communities, creating favorable conditions for functional microbial colonization and biotransformation of flavonoid-based bioactive compounds. The progressive accumulation of bioactive components and flavor metabolites during the freshening-drying-aging process is closely linked to microbial metabolism. Correlation analyses identified *Aspergillus* and *Wallemia* as key functional fungi influencing CRP quality formation.

This study advances beyond traditional research paradigms limited to the drying-aging phase by establishing a comprehensive research framework encompassing the entire freshening-drying-aging continuum. These findings not only provide a scientific basis for standardizing CRP processing but also propose novel strategies for optimizing aging protocols and quality regulation of traditional medicinal-edible products. The integration of microbial dynamics with chemical evolution offers practical insights to promote standardized development in the CRP industry, highlighting its dual significance in advancing fundamental research and industrial applications.

The following are the supplementary data related to this article.

Supplementary material Supplementary material

## CRediT authorship contribution statement

**Yuan Hu:** Writing – original draft, Methodology, Investigation, Conceptualization. **Dailing Hu:** Methodology, Investigation. **Lei Yin:** Methodology, Data curation. **Zhi Deng:** Software, Resources, Methodology. **Yingying Cheng:** Investigation, Formal analysis, Data curation. **Hanxi Li:** Methodology, Data curation. **Fu Wang:** Writing – review & editing, Validation, Investigation. **Youping Liu:** Writing – review & editing, Project administration, Investigation.

## Declaration of competing interest

The authors declare that they have no known competing financial interests or personal relationships that could have appeared to influence the work reported in this paper.

## Data Availability

Data will be made available on request.
