# Supplementary material for: Dynamic profiling of bioactive compounds, flavor metabolites, and quality-related microorganisms during the freshening-drying-aging process of citri reticulatae pericarpium: Implications for quality formation mechanisms
Source: Food Chem X. 2025 Aug 8;30:102906. doi: 10.1016/j.fochx.2025.102906 (PMC12358647; doi:10.1016/j.fochx.2025.102906)
Supplement: Supplementary material [file mmc5.docx]

**Figure S1 Petal diagram.** Each petal in the petal diagram represents one (group) of samples, different colours represent different samples (groups), the CORE numbers in the middle represent the number of OTUs common to all samples, and the numbers on the petals represent the number of OTUs specific to that sample (group).

**Figure S2 Plot of changes in diversity index for each group of samples.** (A) The index of Shannon and Simpson; (B) The index of Chao1and ACE.

**Figure S3 UPGMA clustering tree based on Weighted Unifrac distance.**

**Figure S4 Correlation analysis of main volatile components and microorganisms in the process of freshening-drying-aging of CRP**
